# Clinical Outcomes of Regenerative Orthobiologic Treatments in Hip Avascular Necrosis: A Systematic Review and Meta-Analysis

**DOI:** 10.7759/cureus.110345

**Published:** 2026-06-06

**Authors:** Karun Jain, Velkuru Sai Karthik, Ajay Kumar Rajput, Hizam Naseef, Jujhar Singh, Aishwarya Singh

**Affiliations:** 1 Department of Orthopedics and Trauma, Pushpanjali Medical Centre, New Delhi, IND; 2 Department of Orthopedics, Sai Ram Hospitals, Khammam, IND; 3 Department of Orthopedics, Uttar Pradesh University of Medical Sciences, Saifai, IND; 4 Department of Orthopedics, Bava Memorial Hospital, Malappuram, IND; 5 Department of Sports Injury Center (SIC), Guru Gobind Singh Indraprastha University (GGSIPU), Vardhman Mahavir Medical College (VMMC) and Safdarjung Hospital, New Delhi, IND; 6 Department of Pathology, Shri Guru Ram Rai Institute of Medical and Health Sciences, Dehradun, IND

**Keywords:** avascular necrosis, bone marrow, hip, orthobiologics, stem cells

## Abstract

Hip avascular necrosis is an unstable disease that mostly affects younger patients and is likely to cause collapse of the femoral head, eventually resulting in total hip arthroplasty in spite of efforts put into hip-preservation therapy. The use of regenerative orthobiologic therapies, such as bone marrow-derived cell therapies and platelet-rich plasma, is becoming more popular as it may improve biological repair and retard disease progression. This review evaluated the clinical outcomes of regenerative orthobiologic treatments in hip avascular necrosis, with a focus on conversion to total hip arthroplasty. A systematic review and meta-analysis were conducted according to Preferred Reporting Items for Systematic Reviews and Meta-Analyses (PRISMA) 2020 guidelines. PubMed, Scopus, and the Virtual Health Library were searched from inception to December 2025. Comparative studies involving adult patients treated with regenerative orthobiologics were included. The primary outcome was conversion to total hip arthroplasty, which was evaluated with the help of a random-effects model with risk ratios and 95% confidence intervals. Thirteen studies were included. The primary analysis of cell-based therapies demonstrated a significant reduction in total hip arthroplasty conversion (RR 0.53, 95% CI 0.32-0.86) with moderate heterogeneity (I^2 ^= 66.5%). The secondary analysis of other regenerative interventions also showed a significant reduction (RR 0.43, 95% CI 0.22-0.85) and had no heterogeneity (I^2^ = 0.0%). Regenerative orthobiologic treatments were associated with improved hip preservation and may delay progression to total hip arthroplasty, particularly in early-stage disease.

## Introduction and background

Hip avascular necrosis or avascular necrosis of the femoral head (AVNFH) is a progressive and disabling disorder characterized by the impaired circulation of blood to the femoral head, resulting in ischemia of the bone, its structural degeneration, and subsequent joint damage [1]. Individuals who are in their third to fifth decades of age are most susceptible to the disease, and thus, the disease poses a great functional and socioeconomic burden [2]. Patients come in clinically with pain in the hips, limited movement, and progressive impairment in the joint functions, which have a significant impact on quality of life [3]. When not treated, it often leads to the collapse of the femoral head and secondary osteoarthritis, which eventually requires surgical intervention [1]. Clinically, early or pre-collapse disease refers to a stage in which the femoral head has not yet lost its spherical contour, whereas post-collapse disease indicates structural failure of the femoral head. This distinction is important because biologic treatments are expected to work best before irreversible mechanical collapse occurs.

Hip avascular necrosis is a significant indication of total hip arthroplasty (THA) all over the world, especially in young and active populations, in whom long-term survival of implants is a problem [4]. Different hip preservation surgeries, such as core decompression and femoral osteotomies, have been used at the early disease stages to postpone the progression of the disease [5]. Nevertheless, such traditional interventions prove to have mixed effectiveness and are frequently ineffective in the prevention of the occurrence of the femoral head collapse, particularly in situations when the necrotic lesion size is larger or the disease progression is advanced [6]. As a result, hip avascular necrosis is still poorly managed, and thus, there is a necessity for more efficient and biologically guided treatments [7].

Regenerative orthobiologic therapies have received significant interest despite being a relatively recent field in the past few years as a disease-modifying intervention. Such methods involve the application of bone marrow-derived cells, mesenchymal stem cells, and platelet-rich plasma (PRP), among others, which aim to improve the natural healing ability of bone tissue [8]. The pathophysiology behind this is stimulation of osteogenesis, enhancement of angiogenesis, and regulation of the local microenvironment to support tissue repair [9]. There are growing signs that these biologic approaches are capable of enhancing clinical results and slowing the rate of disease progression when applied as supplements to traditional practice, like core decompression [10]. In addition, tissue engineering and biologic augmentation have opened up the treatment options of hip avascular necrosis and provide promising alternatives to purely mechanical procedures [11].

Although these improvements have been achieved, the clinical efficacy of regenerative orthobiologics remains an issue of debate. The current literature is diverse regarding the choice of patients, intervention strategies, and outcome indicators, thus producing heterogeneous and in some cases conflicting results [3]. There are reports with better hip survival and functional outcomes; however, other reports do not show any significant improvements compared to traditional treatments [6]. Also, the relative performance of various orthobiologic modalities, such as cell-based therapies and PRP-based therapies, has not been clearly determined. Such inconsistency leads to the necessity of a unified synthesis of existing evidence [11].

Even though many studies have explored the regenerative orthobiologic therapies in hip AVNFH, the data are still not consolidated and have not been reported in a standard manner. Good-quality meta-analyses with a direct comparison of various orthobiologic approaches and assessment of clinically meaningful outcomes are scarce. In particular, the conversion to the total hip arthroplasty, which can be referred to as the failure of the treatment and the progression of the disease, has not been analyzed uniformly across the studies. Therefore, the role of orthobiologics in the treatment of AVNFH should be determined through a systematic analysis of the outcomes, which are clinically relevant.

Objectives of the review

This review was purposed to assess the clinical outcomes of regenerative orthobiologic therapy of patients having AVNFH. Specifically, the effect of such interventions on the delay of the disease progression was the parameter that the given review was determined to evaluate, and conversion to arthroplasty of a total hip was chosen as one of the primary indicators of the treatment failure. It also tried to compare the results of cell-based orthobiologics to those of other regenerative approaches to give a better picture of their relative clinical effectiveness.

## Review

Methodology

Search Strategy

The present systematic review and meta-analysis were carried out based on the Preferred Reporting Items for Systematic Reviews and Meta-Analyses (PRISMA) 2020 guidelines [10]. PubMed, Scopus, and the Virtual Health Library (VHL) were searched from database inception to December 2025. The search combined controlled vocabulary and free-text terms related to AVNFH, regenerative orthobiologics, stem cells, bone marrow-based therapies, platelet-rich plasma, disease progression, and total hip arthroplasty. Boolean operators (AND/OR) were used to combine disease-, intervention-, and outcome-related terms. Table 1 demonstrates the search plan of the individual database in detail. 

**Table 1:** Search strategy VHL: Virtual Health Library

Database	Search terms
PubMed	(("osteonecrosis of femoral head"[tw] OR "avascular necrosis"[tw] OR "femoral head necrosis"[tw]) AND ("stem cells"[tw] OR "bone marrow"[tw] OR "mesenchymal stem cells"[tw] OR "platelet rich plasma"[tw] OR "PRP"[tw] OR "orthobiologics"[tw]) AND ("core decompression"[tw] OR "treatment"[tw]) AND ("outcome"[tw] OR "total hip arthroplasty"[tw] OR "progression"[tw]))
Scopus	(TITLE-ABS-KEY ("osteonecrosis" OR "avascular necrosis") AND TITLE-ABS-KEY ("stem cells" OR "bone marrow" OR "PRP" OR "orthobiologics") AND TITLE-ABS-KEY ("core decompression" OR "treatment") AND TITLE-ABS-KEY ("outcome" OR "arthroplasty" OR "progression"))
VHL	Field 1 (Title, abstract, subject): ("osteonecrosis" OR "avascular necrosis") AND ("stem cells" OR "bone marrow" OR "PRP" OR "orthobiologics") AND ("core decompression" OR "treatment") AND ("outcome" OR "arthroplasty" OR "progression")

Eligibility Criteria

Studies were selected according to predefined inclusion and exclusion criteria covering population, intervention, outcome, study design, and data availability (Table 2).

**Table 2:** Inclusion and exclusion criteria THA: total hip arthroplasty; AVNFH: avascular necrosis of the femoral head

Domain	Inclusion criteria	Exclusion criteria
Population	AVNFH in adult, diagnosed patients	Pediatric populations, animal studies, or mixed populations without separable adult data
Intervention	Regenerative orthobiologic (stem cells, bone marrow-derived, or platelet-rich plasma) with or without core decompression.	Studies not involving orthobiologic interventions or evaluating unrelated treatments
Outcome	Studies reporting clinical outcomes, including progression of disease or conversion to total hip arthroplasty	Studies not reporting relevant clinical outcomes or lacking THA/progression data
Study design	Randomized controlled trials and comparative observational studies	Case reports, reviews, editorials, conference abstracts, and single-arm studies
Data requirements	Studies providing extractable numerical data for effect size calculation	Studies lacking sufficient quantitative data for analysis

Study Selection

All records were then uploaded to the system of reference management, and duplicates were removed. Two independent reviewers screened titles and abstracts on the relevance criteria, and a full-text assessment of potentially eligible studies was made. The resolutions to differences were achieved through agreement and discussion.

Data Extraction

Data were extracted independently using a standardized data collection form. Extracted variables included author, year of publication, sample size, patient demographics, intervention, comparator, follow-up duration, and reported clinical outcomes. Outcomes included disease progression and conversion to THA, with THA conversion prioritized for quantitative synthesis because it was the most consistently extractable endpoint.

Risk of Bias Assessment

The quality of the methodology of the included studies was checked through the Cochrane Risk of Bias 2 (RoB 2) tool [11] of randomized controlled trials and the Newcastle-Ottawa Scale (NOS) [12] of observational studies.

Statistical Analysis

A random-effects model was used to account for inter-study variability. The primary clinical endpoint was conversion to THA, selected as a clinically meaningful measure of disease progression and treatment failure. Effect estimates were reported as risk ratios (RRs) with 95% confidence intervals (CIs). Between-study heterogeneity was assessed using the I² statistic and Cochran’s Q test. Publication bias was evaluated using Egger’s regression test, and leave-one-out sensitivity analysis was performed to assess the robustness of pooled estimates.

Inverse-variance weighting was applied under the random-effects model, and heterogeneity was interpreted according to the magnitude of the I² statistic. Statistical significance was defined as p < 0.05. A formal certainty-of-evidence assessment using GRADE was not performed, which should be considered when interpreting the strength of the pooled evidence.

Subgroup analysis and meta-regression were not performed because of the limited number of studies and substantial clinical heterogeneity in intervention protocols, disease stage, and study design, which could have reduced the reliability of such analyses.

Results

Study Selection

**Figure 1:**
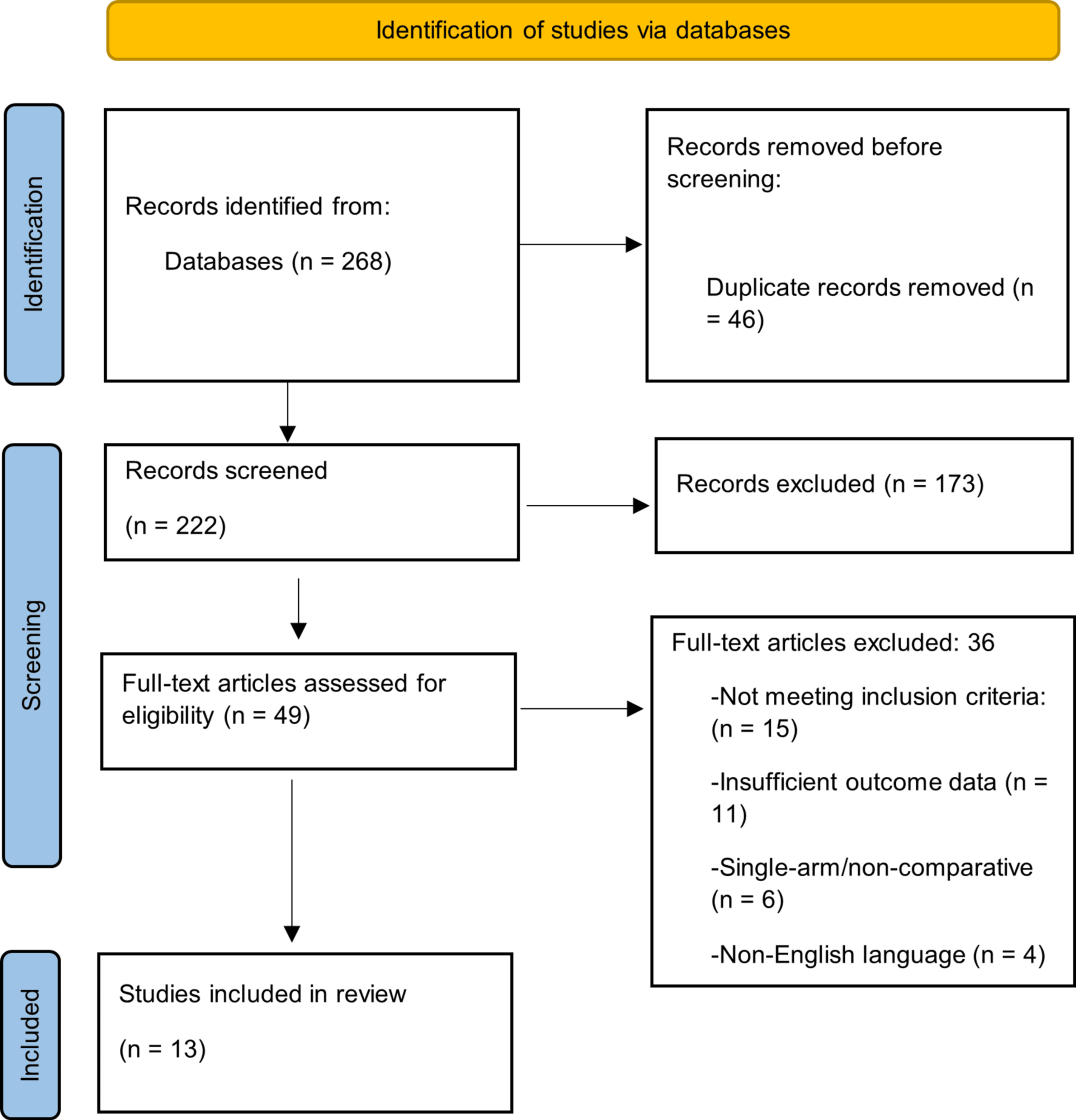
PRISMA flow diagram of study selection PRISMA: Preferred Reporting Items for Systematic Reviews and Meta-Analyses

The database search identified 268 records. After removal of 46 duplicates, 222 records were screened by title and abstract, of which 173 were excluded. Forty-nine full-text articles were assessed for eligibility. Of these, 36 were excluded because they did not meet the inclusion criteria (n = 15), lacked relevant outcome data (n = 11), used a single-arm or non-comparative design (n = 6), or were published in a non-English language (n = 4). Thirteen studies were included in the systematic review and quantitative synthesis (Figure 1).

Study Characteristics

Thirteen studies, including randomized controlled trials and comparative observational studies, evaluated regenerative orthobiologic interventions in AVNFH (Table 3). Interventions included bone marrow-derived cell therapies, mesenchymal stem cells, PRP, osteoblastic cell therapy, and biologic graft augmentation. Nine studies were included in the primary cell-based orthobiologics analysis, and four studies were included in the secondary regenerative intervention analysis. Across studies, conversion to THA was used as the primary pooled clinical endpoint because it reflects clinically relevant failure of hip-preserving treatment and disease progression.

**Table 3:** Characteristics of included studies BMMCs: bone marrow mononuclear cells; CD: core decompression; BMMSCs: bone marrow-derived mesenchymal stem cells; RCT: randomized controlled trial; MSCs: mesenchymal stem cells; BMC: bone marrow concentrate; BMAC: bone marrow aspirate concentrate; BBC: bone marrow buffy coat; PRP: platelet-rich plasma; BG: bone graft

Study	Year	Study Design	Intervention	Comparator	Follow-up
Gangji et al. [13]	2011	Prospective controlled	BMMCs + CD	CD alone	60 months
Zhao et al. [14]	2012	RCT	BMMSCs + CD	CD alone	60 months
Rastogi et al. [15]	2013	Prospective	MSCs	Bone marrow	24 months
Ma et al. [16]	2014	Double-blind RCT	Buffy coat + CD	CD	24 months
Tabatabaee et al. [17]	2015	Comparative	Stem cells + CD	CD	24 months
Cruz-Pardos et al. [18]	2016	Retrospective	BMC + CD	CD	~45 months
Hauzeur et al. [19]	2018	RCT	BMAC + CD	CD	24 months
Hernigou et al. [20]	2018	Prospective paired	Cell therapy + CD	CD	30 years
Kang et al. [21]	2018	Matched-pair	BMMSCs + CD	CD	4.3 years
Hauzeur et al. [22]	2020	RCT	Osteoblastic cells	BMAC	36 months
Li et al. [23]	2021	Randomized	BBC + graft	Graft only	24 months
Aggarwal et al. [24]	2021	Randomized	PRP + CD	CD	4.5–6 years
Xu et al. [25]	2024	Retrospective	PRP + CD + BG	CD + BG	5 years

Cell-Based Orthobiologics Results

**Figure 2:**
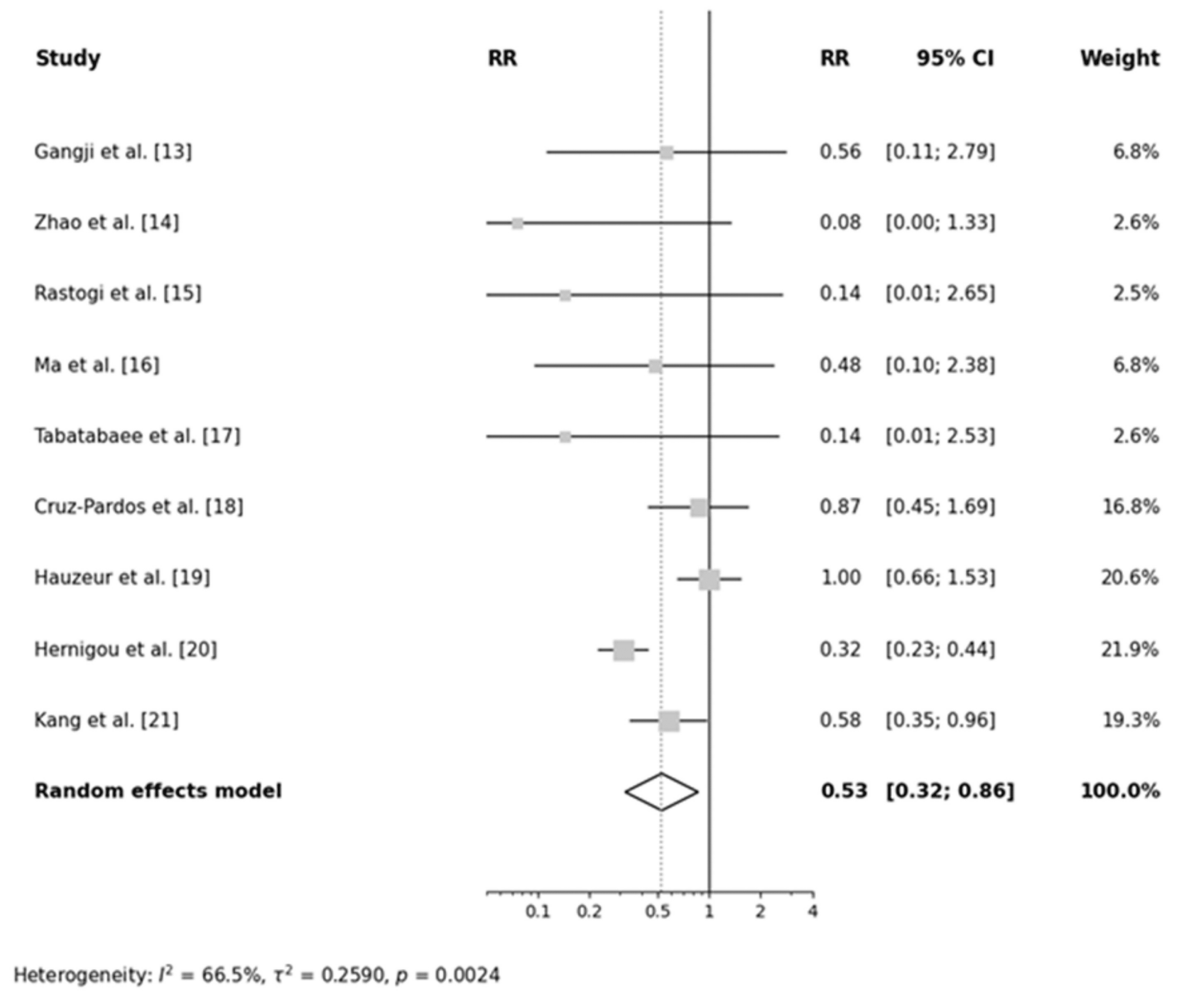
Forest plot showing the effect of cell-based orthobiologics on total hip arthroplasty conversion RR: risk ratio; CI: confidence interval; I^2^: heterogeneity statistic; τ^2^: between-study variance

Nine studies were considered in the primary meta-analysis that determined the impact of cell-based orthobiologic interventions on clinical outcomes, with the primary goal determined as conversion to THA. The pooled analysis showed that cell-based therapies were linked with a 1.54-fold decrease in THA conversion with a pooled RR of 0.53 (95% CI: 0.32-0.86). There was moderate heterogeneity between studies (I² = 66.5%, τ² = 0.259, p = 0.0024). This heterogeneity was likely related to differences in disease stage, lesion severity, cell source and processing method, adjunctive use of core decompression, follow-up duration, and study design. These factors may have affected baseline progression risk and the observed magnitude of treatment benefit across studies (Figure 2).

Regenerative Comparative Studies Results

**Figure 3:**
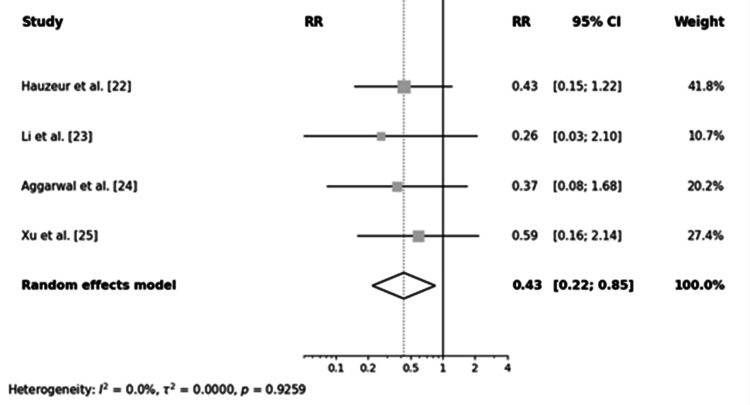
Forest plot showing the effect of regenerative interventions on total hip arthroplasty conversion RR: risk ratio; CI: confidence interval; I²: heterogeneity statistic; τ²: between-study variance

The secondary analysis included four studies evaluating other regenerative orthobiologic strategies, including PRP-based interventions, osteoblastic cell therapy, and biologic graft augmentation. These interventions were associated with a significant reduction in THA conversion, with a pooled RR of 0.43 (95% CI: 0.22-0.85). Heterogeneity was negligible (I² = 0.0%, τ² = 0.000, p = 0.9259), indicating consistent treatment effects across the included studies (Figure 3).

Risk of Bias Assessment

The included studies had an overall low-to-moderate risk of bias (Table 4). Randomized controlled trials were generally judged to have low risk of bias, reflecting acceptable methodological rigor in randomization and outcome assessment. Observational and comparative studies were most often judged to have moderate risk of bias, mainly because of inherent limitations such as lack of blinding, retrospective design, and potential selection bias.

**Table 4:** Risk of bias assessment of included studies RCT: randomized controlled trial; NOS: Newcastle–Ottawa Scale; RoB 2: Cochrane Risk of Bias 2 tool However, the test conducted by Egger [26] did not show significant publication bias in the primary analysis (p = 0.746) and secondary analysis (p = 0.377), but the interpretation should be limited as there are a few studies. A leave-one-out sensitivity analysis indicated that there is no significant difference in the pooled estimates in either analysis.

Study	Design	Tool Used	Overall Judgment
Gangji et al. [13]	Prospective controlled	NOS	Moderate
Zhao et al. [14]	RCT	RoB 2	Low
Rastogi et al. [15]	Prospective	NOS	Moderate
Ma et al. [16]	Double-blind RCT	RoB 2	Low
Tabatabaee et al. [17]	Comparative	NOS	Moderate
Cruz-Pardos et al. [18]	Retrospective	NOS	Moderate
Hauzeur et al. [19]	RCT	RoB 2	Low
Hernigou et al. [20]	Paired comparative	NOS	Moderate
Kang et al. [21]	Matched-pair	NOS	Moderate
Hauzeur et al. [22]	RCT	RoB 2	Moderate
Li et al. [23]	Randomized	RoB 2	Moderate
Aggarwal et al. [24]	Randomized	RoB 2	Low
Xu et al. [25]	Retrospective	NOS	Moderate

Discussion

Interpretation of Main Findings

This meta-analysis summarized comparative evidence on regenerative orthobiologic therapies for AVNFH and found that these interventions were associated with reduced progression to THA. The primary cell-based orthobiologics analysis showed a significant reduction in THA conversion with moderate heterogeneity, whereas the secondary analysis of other regenerative strategies also showed a significant benefit with negligible heterogeneity. Overall, these findings suggest that biologically enhanced hip-preserving interventions may improve clinically meaningful outcomes, particularly when used before permanent structural failure.

The best signal in the current review consisted of those studies assessing bone marrow-derived cellular therapies. Gangji et al. have indicated long-term efficacy of the use of autologous bone marrow cell implantation at five years, which also confirms the idea that biologic enhancement can increase the hip survival in the absence of trauma in osteonecrosis [13]. This observation was also reinforced by Zhao et al., who demonstrated positive results when using cultured bone marrow-derived mesenchymal stem cells in early-stage disease [14]. Similarly, Rastogi et al. discovered that encouraging early clinical results were correlated with intralesional autologous mesenchymal stem cells, but the research was initial, and the sample was small [15]. Ma et al. in a randomized study proved that the efficacy of autologous bone marrow buffy coat grafting when used with core decompression was better than the efficacy of decompression alone, which supports the use of marrow-derived biologic augmentation as a significant option in therapy [16].

Influence of Disease Stage and Biologic Platform

The other studies in this review also indicate that the effect of treatment could vary depending on the stage of the disease, as well as the biologic platform. Tabatabaee et al. demonstrated that core decompression and concentrated autologous bone marrow stem cell injection improved the outcomes in early disease, which should be correlated to the principle that regenerative therapies are most likely to benefit before there is significant collapse [17]. Cruz-Pardos et al. also confirmed a more neutral mid-term result of standard practice, which pointed out that the advantages of marrow concentrate might be less obvious in unselected real-life populations [18]. This stage-dependence was further demonstrated in the study conducted by Hauzeur et al., in which, in the case of stage III osteonecrosis, no such stage III improvements were found with the use of autologous bone marrow concentrate, indicating that the use of biologic supplementation alone might not be sufficient in overcoming the advanced mechanical failure [19].

Therefore, the pooled treatment effect may have been influenced by the proportion of pre-collapse and post-collapse hips included across studies. Studies enrolling predominantly early-stage or pre-collapse cases may have contributed more strongly to the apparent benefit of biologic augmentation, whereas studies including advanced or post-collapse disease may have attenuated the pooled effect because structural failure is less biologically reversible. Conversely, Hernigou et al. described lasting better results on long-term experiments using cell therapy compared to contralateral decompression, which supports the importance of biologic intervention to be utilized in properly chosen hips [20].

Additional included studies further support the role of cell-based and non-cellular biologic augmentation, although the magnitude of benefit varied by intervention type and comparator. Kang et al. observed a better clinical efficacy of bone marrow mesenchymal stem cell implantation over that of simple core decompression, and this finding, in addition, lends credence to the use of cell-based adjuvants to preserve the hip [21]. Nevertheless, not every regenerative strategy seems to be the same. Later, Hauzeur et al. assessed the osteoblastic cell therapy, which did not show explicit better results than compared to the comparator treatment, indicating that adding more biologic complexity is not always associated with improved clinical outcomes [22]. Li et al. demonstrated that in the case of structural support and biologic stimulation, autologous bone marrow buffy coat combined with angioconductive bioceramic rod grafting had a positive impact on short-term outcomes [23]. Likewise, Aggarwal et al. found that PRP instillation with core decompression enhanced the function and delayed progression of mild disease cases, suggesting that non-cellular orthobiologics still can provide a significant clinical benefit [24]. The retrospective design was also found by Xu et al. to yield positive results when PRP is used with decompression and bone grafting, but should be cautiously interpreted [25].

Clinical Relevance of THA Conversion

The current results are clinically applicable since THA conversion is not just a radiographic outcome but an expedient outcome of treatment failure. However, THA conversion may also be influenced by non-biological factors, including surgeon- and institution-specific thresholds for arthroplasty, patient preference, healthcare access, and differences in follow-up duration across studies. Therefore, while THA conversion is a clinically meaningful endpoint, the pooled treatment effect should be interpreted with recognition that it may partly reflect variation in surgical decision-making patterns and follow-up opportunity rather than disease biology alone. Delays of arthroplasty in young and active patients are a key goal since the risk of revision is very high, and the implant burden over the long-term is very high.

Not even contemporary bone-saving arthroplasty tactics can eradicate the consequences of early THA in the long run in osteonecrosis [27]. It may be functional in AVNFH, yet the population of patients who undergo THA is typically younger than in the case of degenerative arthritis, and this makes hip preservation especially appealing [28]. This is of more concern in subgroups that are medically complex, like in the sickle cell disease, where arthroplasty may be technically challenging, and there may be more risk during perioperative conditions [29]. It is on these grounds that even without completely reversing disease biology, an intervention that will reduce THA conversion may still be of great clinical benefit.

Implications for Hip Preservation

The outcomes also confirm the general idea that the core decompression effect can be increased by orthobiologic adjuvants instead of being substituted. The recent studies in the field of arthroplasty and hip preservation still aim to underline the role of stage-appropriate treatment and biological optimization within AVNFH [30]. An earlier assessment of orthobiologic adjuvants has also reached a similar conclusion that biologic augmentation can be used to enhance hip preservation, but the quality and consistency of the evidence are inconsistent [31]. A recent systematic review and meta-analysis of core decompression combined with regenerative therapy in early-stage osteonecrosis similarly reported potential benefit while emphasizing heterogeneity in regenerative modalities, disease-stage definitions, and outcome reporting [32].

Limitations and Future Directions

This study has several limitations. First, the studies included were heterogeneous in terms of the type of intervention, processing methods, level of disease, and follow-up period. Second, the research encompassed both randomized and observational comparative studies, which can potentially cause methodological variation even though risk-of-bias assessment was done separately. Third, the THA conversion endpoint was the most extractable endpoint, and pain scores, functional scores, and radiographic progression were not consistently reported across the studies. Fourth, the number of studies used in testing publication bias was small, especially in the secondary analysis.

Future studies need to emphasize adequately powered randomized trials, where the disease stage is defined, and both the functional and radiographic outcomes are uniformly reported and followed up with longer follow-up. It would be especially valuable to compare the cell-based therapies, PRP-based strategies, and mixed approaches involving scaffold-biologic approaches. An improved stratification with lesion size and pre-collapse versus post-collapse status could also play a role in determining the patients who are most likely to recover with orthobiologic treatment.

## Conclusions

The regenerative orthobiologic interventions seem to result in hip preservation in the AVNFH, especially when it is performed before the structural failure. In the comparative studies, cell-based treatments and other regenerative methods were linked with less conversion of THA, which showed their capability of being used as a supplement to hip-preserving surgeries. Bone marrow-derived cellular interventions showed the most reliable benefits, and PRP-based and other biologic methods also proved to have positive outcomes in a chosen situation. These results suggest that biological augmentation can enhance the efficacy of traditional decompression to overcome the defective regenerative condition of osteonecrosis. The inconsistency in intervention procedures, disease progression and the type of studies designed, however, does not allow for defining one optimal strategy. Regenerative orthobiologics is an innovative method of disease preventive treatment and slowing down of disease progression. More properly designed studies with standardized outcomes are needed to determine their relative efficacy and to determine their place in clinical practice.
